# Observing Spring and Fall Phenology in a Deciduous Forest with Aerial Drone Imagery

**DOI:** 10.3390/s17122852

**Published:** 2017-12-08

**Authors:** Stephen Klosterman, Andrew D. Richardson

**Affiliations:** 1 Department of Organismic and Evolutionary Biology, Harvard University, Cambridge, MA 02138, USA; Andrew.Richardson@nau.edu; 2 School of Informatics, Computing and Cyber Systems, Northern Arizona University, Flagstaff, AZ 86011, USA; 3 Center for Ecosystem Science and Society, Northern Arizona University, Flagstaff, AZ 86011, USA

**Keywords:** phenology, Harvard Forest, leaf color, plant area index, drone, UAV

## Abstract

Plant phenology is a sensitive indicator of the effects of global change on terrestrial ecosystems and controls the timing of key ecosystem functions including photosynthesis and transpiration. Aerial drone imagery and photogrammetric techniques promise to advance the study of phenology by enabling the creation of distortion-free orthomosaics of plant canopies at the landscape scale, but with branch-level image resolution. The main goal of this study is to determine the leaf life cycle events corresponding to phenological metrics derived from automated analyses based on color indices calculated from drone imagery. For an oak-dominated, temperate deciduous forest in the northeastern USA, we find that plant area index (PAI) correlates with a canopy greenness index during spring green-up, and a canopy redness index during autumn senescence. Additionally, greenness and redness metrics are significantly correlated with the timing of budburst and leaf expansion on individual trees in spring. However, we note that the specific color index for individual trees must be carefully chosen if new foliage in spring appears red, rather than green—which we observed for some oak trees. In autumn, both decreasing greenness and increasing redness correlate with leaf senescence. Maximum redness indicates the beginning of leaf fall, and the progression of leaf fall correlates with decreasing redness. We also find that cooler air temperature microclimates near a forest edge bordering a wetland advance the onset of senescence. These results demonstrate the use of drones for characterizing the organismic-level variability of phenology in a forested landscape and advance our understanding of which phenophase transitions correspond to color-based metrics derived from digital image analysis.

## 1. Introduction

Phenology, the study of recurrent biological events, has been a focus of plant science for centuries [1]. Phenology responds to interannual and spatial variability in environmental conditions, particularly temperature [2,3], and also mediates key ecosystem functions, including carbon assimilation and evapotranspiration [4,5,6,7,8]. As global environmental change becomes an increasingly public issue, the value of plant phenology as a sensitive indicator of the effects of change has kindled interest in creating and interpreting phenology records, such as those from digital repeat photography. Tower- or building-mounted phenocams, typically located at positions just above the canopy [9,10,11], preserve a valuable visual record of vegetation phenology in forests and other ecosystems. “Near-surface” methods such as phenocams complement the phenology records of satellite remote sensing, which extensively observe entire landscapes, but at a spatial resolution that typically makes it impossible to discern individual plants [12]. Unlike other near-surface methods based on radiometric sensors, e.g., [13,14], images from phenocams have the advantage that they can be visually interpreted by direct examination.

With phenocam imagery, it is also possible to objectively quantify phenology from time series of vegetation “greenness” or “redness”. Discrete phenophase transition dates can be derived from those time series, using techniques developed for analyzing satellite remote sensing data [15,16]. These methods have been successfully applied to the evaluation of satellite remote sensing phenology products [17,18] and exploration of the connections between ecosystem function, e.g., carbon assimilation, and the greenness metrics of phenology [5,6]. However the oblique angle of tower-mounted cameras has been suggested to result in biased estimations of the timing of canopy maturity, compared to the vertically integrated plant area index (PAI), as well as in situ observations of leaf expansion [19]. Phenocam imagery does make it possible to easily integrate across multiple organisms in the camera field of view and thus characterize landscape-level phenology. But, in mixed-species stands, these estimates may be biased because the organisms located closest to the camera dominate the field of view and hence are over-represented, while more distant organisms are under-represented.

Drones, also called UAVs, open up an exciting new possibility for the near-surface remote sensing of plant phenology. With drones, researchers can obtain aerial photography with a similar spatial resolution to tower-mounted phenocams, but at the landscape scale, similar to satellite remote sensing. Compared to traditional aircraft, drones can be operated at a fraction of the cost, making more frequent observations feasible. Additionally, using photogrammetry techniques with drone images facilitates significant advances over tower-mounted cameras. Orthomosaics simulate an undistorted nadir perspective of the canopy, with a consistent spatial resolution over landscapes [20]. Because of this feature, orthomosaics enable the identification and analysis of larger numbers of individual organisms than is typically possible using tower-mounted camera imagery.

Previous research has begun to explore the potential of drones for the study of forest phenology. The first study to use aerial drone imagery to explore forest structural and color properties demonstrated that the changing color of leaves at different times of the growing season can be quantified by applying digital image processing techniques to georeferenced orthomosaics [20]. Subsequently, researchers leveraged the high spatial resolution of near-surface aerial drone photography to delineate crowns and study the phenology of individual trees. It was found that the color of individual tree crowns could be used to identify their species, based on knowledge of their expected phenological status during different times of the season [21]. In another study, high temporal frequency aerial images taken during springtime were used to show that the timing of budburst of individual trees, observed in situ, appears to coincide with the beginning of an increase in a canopy greenness metric calculated from the images of these trees [22]. In our previous work, which used an earlier year of imagery from the same study site reported here, we used drone imagery to show how landscape scale variance in phenology could be attributed to plant species, and explored the nature of the spatial variability of phenology at different spatial resolutions [23].

Our goal in this study is to extend previous efforts to determine the leaf life cycle events of trees that correspond to digital image analysis metrics of those same trees in aerial drone imagery. We use imagery of the complete growing season to go beyond budburst and consider leaf expansion in spring, as well as color change and abscission in autumn. We also compare metrics derived from aerial images with the progression of PAI, to interpret color indices with reference to canopy structural development. Finally, we examine how contrasting air temperature regimes at microsites within the study area correlate with spatial variation in landscape phenology, after accounting for spatial variance induced by differences in tree species. These results demonstrate the use of drones for observing the complete seasonal cycle of deciduous canopy development at the landscape scale, with a high enough spatial resolution to discern organism-level phenomena.

## 2. Materials and Methods 

### 2.1. Study Site

**Figure 1:**
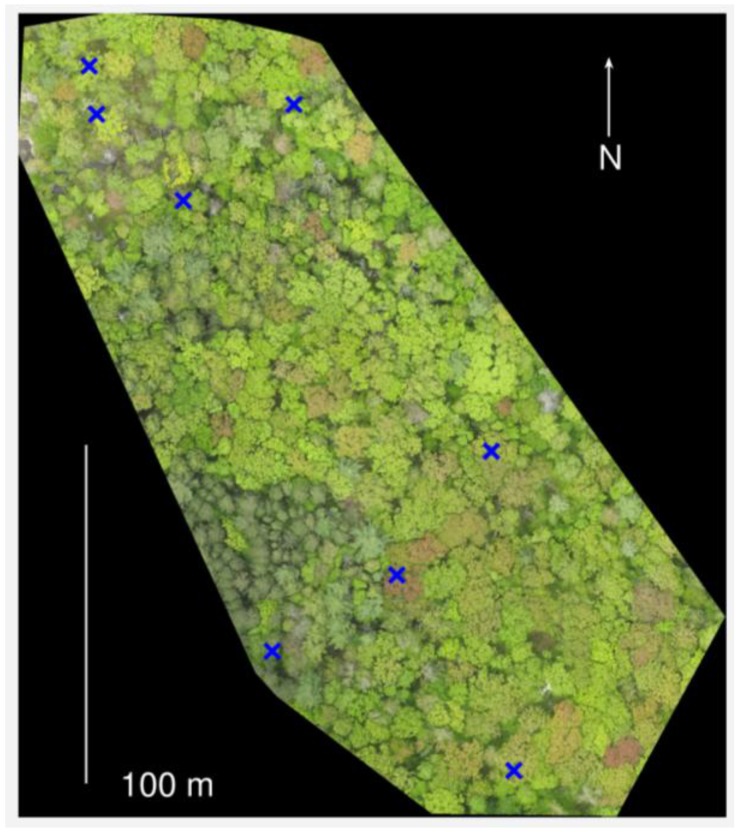
Study area at Harvard Forest on 5/21/17 (DOY 141). Location of microsite temperature loggers indicated as blue “×” symbols.

We conducted our study at Harvard Forest in Petersham, MA, USA (42.5377° N, 72.1715° W). The climate is temperate, with a mean annual precipitation of 110 cm and mean annual temperature of 7.1 °C. The study area is a mixed deciduous-evergreen forest, with some woody wetlands. Deciduous trees in the study area include predominantly red oak (*Quercus rubra*) and red maple (*Acer rubrum*), as well as yellow birch (*Betula alleghaniensis*), American beech (*Fagus grandifolia*), and black oak (*Quercus velutina*). The study area is 2.8 ha in size and contains approximately 1900 trees with a diameter at breast height ≥10 cm (Figure 1).

### 2.2. Drone Image Acquisition and Processing

**Figure 2:**
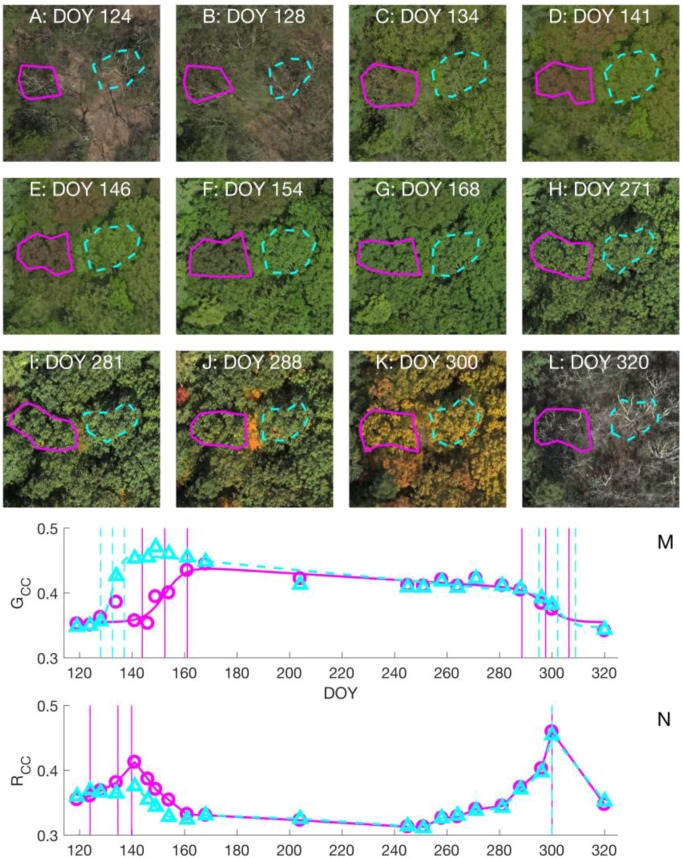
(**A**–**L**): Close-up (30 m by 30 m) of aerial images for a selection of dates from 2015. Each close-up shows the ROIs used to analyze two red oak trees. Solid magenta ROIs were used to calculate solid magenta G_CC_ and R_CC_ values (circles symbols) and curves; similar with the dashed cyan ROIs (triangle symbols.). Images have been equalized to have the same brightness (sum of R, G, and B digital numbers) for visualization purposes in this figure; (**M**,**N**): the resulting G_CC_ and R_CC_ time series (symbols); curve fits or interpolations; and estimated phenophase transition dates (vertical lines). The vertical lines show six annual dates for both G_CC_ time series, while spring dates calculated from R_CC_ series are only shown for the tree with red spring leaves. R_CC_-EOF occurred on the same date for both trees.

We used methods described in an earlier study [23] to obtain and process aerial photography. Briefly, we used a drone (3DR ArduCopter Quad-C Frame, 3D Robotics, Berkeley, CA, USA) equipped with a Canon Powershot A3300 camera (35 mm film equivalent focal length 28 mm, approx. 16 million pixels). We used the same camera and image settings for all flights, and took pictures of a gray reference square (ColorChecker classic, X-rite, Grand Rapids, MI, USA) before each flight. Flight frequency was roughly every five days during spring leaf out and every week during autumn leaf color change, depending on the weather conditions (dates shown in Figure 2). Images were taken at a minimum shutter speed of 1/1000 s, with constant exposure during each flight. The same color balance was used for all acquisition dates, because consistent color balance is necessary for reliable digital camera observations of phenology [11]. We conducted flights at mid-day (between 10 a.m. and 3 p.m.) on either clear or evenly overcast days, and never during periods of variable cloud cover, as exposure was constant during flights. For each imagery acquisition date, we created orthophotos of the study area using about 100 JPEG photos taken with an intervalometer script (Canon Hack Development Kit, http://chdk.wikia.com/wiki/CHDK), with the PhotoScan photogrammetry software (Agisoft, St. Petersburg, Russia), and performed final georeferencing in ERDAS IMAGINE AutoSync (Intergraph, Huntsville, AL, USA). The orthophotos used in this study are available in the Harvard Forest Data Archive [24].

We calculated green chromatic coordinate (G_CC_) [25] and red chromatic coordinate (R_CC_) [11] time series from orthophotos using Matlab (R2017a), to account for changes in scene illumination due to differences in atmospheric conditions or times of day between flights:

G_CC_ = G/(R + G + B), (1)

R_CC_ = R/(R + G + B), (2)

where R, G, and B are the mean red, green, and blue digital numbers, respectively, in regions of interest from image files. Example G_CC_ and R_CC_ time series and additional processing details are available in Figure S1. We note that the phenology signal observed in the G_CC_ and R_CC_ time series of vegetation was approximately 10 times greater in amplitude than the noise observed in the analysis of the gray reference square, which arose due to factors such as varying illumination conditions and the different times of day of flights. We also explored other indices, including G_CC_ + R_CC_, GRVI, ExG, and Hue, as described in the caption of Figure S2, but found the most reliable results from G_CC_ and R_CC_, and used them in our analysis.

Color indices were calculated using different regions of interest (ROIs) depending on the goal of the analysis. For comparison between image-based metrics and in situ observations of individual tree phenology, we drew ROIs around the crowns of the trees that we observed, as shown in Figure 2. For comparison to upward photo-based estimates of PAI, we created ROIs representing the canopy projection of the image field of view from the ground perspective. To examine phenology across the landscape, we used a square grid of 10 m ROIs (grid cells).

### 2.3. Estimating Phenology Dates from Time Series Data

We used curve fitting methods detailed in Klosterman et al. [18] to estimate phenology dates from time series data, including G_CC_ and additional data described below. Sigmoid models are commonly used to approximate the seasonal trajectory of vegetation indices and to facilitate the determination of phenological transition dates. The greendown sigmoid is a variant of the standard dual sigmoid model [16]; a key difference is that the greendown sigmoid allows for a linear decrease in greenness over the course of the growing season. We used the greendown sigmoid model to approximate the seasonal trajectory of G_CC_ for each ROI. Phenology dates were calculated from curve fit parameters by finding the dates of extrema in the curvature change rate (CCR) [15]. We used these methods to calculate dates for the start, middle, and end of canopy development in spring (G_CC_-SOS, G_CC_-MOS, G_CC_-EOS), and similar dates for canopy senescence in fall (G_CC_-SOF, G_CC_-MOF, G_CC_-EOF). We estimated uncertainties using the Jacobian matrix of curve fit parameters to generate Monte Carlo ensembles of phenology dates, and calculated the inner 95% confidence interval of these ensembles.

To estimate phenology dates from R_CC_ time series, we calculated the dates of crossing the 10th, 50th, and 90th percentiles of a linear interpolation of R_CC_ values, while R_CC_ increased to its spring maximum, to determine R_CC_-SOS, R_CC_-MOS, and R_CC_-EOS. We used the date of the maximum redness value in fall to represent R_CC_-EOF. We determined uncertainties as the interval between the nearest two observation dates, or for transitions that occurred on an observation date, as the interval between the midpoints of that date and the nearest two dates.

In addition to these automated curve fitting and interpolation approaches, we also visually identified the day of the first observable leaves in aerial images of the individual trees discussed below in Section 2.4.1.

### 2.4. In Situ Measurements

We identified eight microsites within the study area based on earlier results, using imagery from a previous year at this site. We had previously found that species composition explained most of the spatial variance in phenology across the Harvard Forest landscape, using a multiple linear regression [23]. However, we identified locations within the study area where regression residuals were relatively large; these were the same areas in regressions using both the 2013 data from the previous study and the 2015 data from the present study. We located microsites in these places, as well as locations where residuals were relatively small, to better understand the drivers of phenology throughout the study area. To provide context for the image-derived phenological transition dates, we made direct observations of phenology on individual trees, and measured canopy-level PAI using digital cover photography. To characterize the microsite environment, we measured air temperature.

#### 2.4.1. Direct Observation of Trees

We observed the phenological status of 30 trees spread across the eight microsites within the study area during each drone photography acquisition date (Figure 1, three to five trees per microsite), and one additional date when the drone was not flown due to weather constraints (5/17/15, DOY 137). Similar to a protocol of established observations of tree phenology at Harvard Forest [26], we estimated the percentage of buds burst on trees, and average leaf size (length) as a percentage of mature leaf size. In autumn, we estimated the percentage of leaves that had changed color due to senescence, and that had fallen. From these observations, we created time series using linear interpolation, and found the point in time corresponding to the deciles of phenological status for each phenology event: day of year for 10%, 20%, … 90% of budburst, leaf size, leaf color, and leaf fall. We calculated uncertainties in the same way used for R_CC_ time series.

We determined the leaf life cycle events corresponding to image-derived metrics of individual tree ROIs by finding the decile of progress in a specific event that had the lowest RMSD with respect to a given metric across the 30 trees under observation. In other words, we compared all deciles (10%, 20%, …) of all observed events (e.g., budburst, leaf size) to each of the image-derived transitions (SOS, MOS, …), and identified the decile that had the minimum RMSD across all trees (e.g., 10% budburst was closest to SOS). We also examined correlations of G_CC_ and R_CC_ values on each date with percentages of progress in each life cycle event on the same date, within and across trees. Since color indices and leaf transitions typically trend in the same direction, for example, increasing G_CC_ and leaf size in spring, an ordinary least squares regression may yield spuriously low coefficient standard errors. Therefore, we used an econometric approach to time series regression: heteroscedasticity and autocorrelation consistent (HAC) regression. HAC regression calculates the variances of regression coefficients (i.e., square of standard error, used to calculate regression *p*-value) based on the inferred autocorrelation structure of regression residuals; the coefficient estimates themselves are the same as those of ordinary least squares. We used the ‘hac’ function in Matlab, with pre-whitening at a lag of one time step and the quadratic spectral weighting scheme, as we found that these options led to the most conservative estimates of coefficient variances in a sensitivity analysis [27,28]. We used *t*-tests to see if there were significant differences in regression parameters between trees. We also calculated ordinary least squares regressions of pooled values across all trees.

#### 2.4.2. Digital Cover Photography (DCP)

Upward photos were taken with the same model of camera used for drone photography (non-fisheye lens). We took images manually for upward photos, on the same dates we acquired drone photography, using the same image settings (i.e., automatic exposure), except for white balance, which was set to “auto” for DCP [29]. We used a level to aim the camera directly upward and positioned the camera on the posts holding the temperature data loggers (1 m height, below most mid-canopy trees), in the same orientation on each date. The projected camera field of view had an area of 533 m^2^ in the canopy (at the average Harvard Forest canopy height of 22 m). We estimated PAI from upward photos at each microsite using a freely available DCP software tool obtained from Craig MacFarlane (contact information in references [29,30]). We calculated sigmoid curve fits of PAI values to estimate transition dates as with G_CC_ time series. However, we observed that PAI values were generally stable from June to September, so we used the traditional sigmoid model as opposed to the greendown sigmoid, effectively setting the slope of the summertime greendown to zero. We also directly compared G_CC_ and R_CC_ values with PAI values, by performing HAC regressions at the microsite level, as both PAI and color indices typically trend in the same direction at each microsite. We also performed ordinary least squares regressions of data pooled across microsites for spring and fall, similar to the analysis described in Section 2.4.1.

#### 2.4.3. Air Temperature Measurements and Effects of Microclimate on Phenology

Because the microsite locations included places with the largest residuals in a regression of species composition and phenology dates, we suspected that additional factors may contribute to the timing of microsite phenology. Temperature effects on phenology have been widely studied [18,31,32,33]. Therefore, we recorded air temperatures half hourly from 11 April 2015, approximately one month before the beginning of leaf out, through December 2015, after completion of leaf fall. To do this, we installed a HOBO U23-004 temperature data logger at a 1 m height with an RS-1 radiation shield (Onset Computer Corp, Bourne, MA, USA) at each microsite.

To determine the effect of microclimate on phenology, we correlated spring and fall temperatures with residuals from a statistical model that accounted for the effects of species variation and land cover type, but not microclimate, on the aggregated canopy phenology of 10 m grid cells. A 10 m grid was chosen based on previous analysis, taking into account spatial inaccuracies in the orthophotos and the nature of the species data [23]. More specifically, the model used tree species from a map of all woody stems ≥1 cm diameter at breast height [34]. This map covered 89% of the land area monitored in drone imagery. Woody species composition was determined using all species that appeared in at least 10 of 245 grid cells, and had at least a 20% basal area fraction in any one grid cell. The remaining species were lumped into one predictor to ensure that the fractional species composition of each grid cell summed to 1. Any grid cell that had <50% coverage in the aerial images was eliminated from this analysis, removing partially covered grid cells on the edge of the imaged area and ensuring all data points had adequate spatial representation. We used a no-intercept regression since fractional species compositions are mixture components that sum to 1 [35]. We then calculated the average residual from these spring and fall regressions for the three by three windows of grid cells centered on the microsite locations (30 m by 30 m areas), and regressed these microsite-average residuals on the monthly means of daily minimum, mean, and maximum temperatures for April (MOS) and September (MOF), as temperatures preceding phenology events are commonly used to predict the timing of those events [18,31,32,33]. We note that we used R_CC_-MOS for grid cells with red spring leaves, following a criterion discussed in the Results Section 3.1, as long as the amplitude in R_CC_ was greater than the range of noise observed in the gray reference square (Figure S1). For all other grid cells, we used G_CC_-MOS. We used Bonferroni correction to account for making three temperature comparisons with both phenology transition dates.

## 3. Results

### 3.1. Choice of Color Index in Spring Time

While changes in canopy greenness have commonly been used to track spring budburst and leaf expansion in deciduous forests, we found that in some instances, these processes appeared to be more associated with changes in redness (Figures S2 and S3). For two of the 30 trees we observed in situ, both red oaks, we saw that leaves were various shades of red (pink, orange) in color during leaf expansion (Figure 1, close up in Figure 2D,E, and Figure S4). Additional oak trees, which were not under in situ observation at the microsites, can also be seen to display red spring leaves in Figure 1. We note that leaves higher in the canopy, i.e., those on the top of the crown and most visible from an aerial view, often appeared to be redder than leaves closer to the ground (Figure S4).

For trees with red spring leaves, the springtime G_CC_ profiles showed a delayed increase compared to trees with green spring leaves, including nearby conspecifics that we observed to have nearly the same leaf expansion phenology (Figure 2M,N). However, the springtime R_CC_ time series exhibited a marked peak for trees with red spring leaves. Then, as R_CC_ decreased, G_CC_ increased as leaves became greener in late spring (Figure 2F,G). We found that for these trees, the springtime amplitude in R_CC_ (the increase from the dormant value to the spring maximum) was more than 45% of the spring amplitude in G_CC_ (46% and 64% for the two trees), while for all other trees, it was less than 35%. The springtime increase in R_CC_ was closer in time to observed leaf expansion than G_CC_, as well as a range of other color indices we considered (G_CC_ + R_CC_, GRVI, ExG, Hue, Figure S2). We also note that the leaves of oak trees with red spring leaves had no apparent color difference in autumn from those of trees with green spring leaves (Figure 2K).

### 3.2. Leaf Life Cycle Events of Trees: Correspondence to Image Metrics

We determined the leaf life cycle events corresponding to image metrics by finding the closest decile of life cycle event (i.e., 10% budburst) to each metric (SOS, MOS, etc.) for the 30 trees under observation. Following our observation that R_CC_ was a better indicator of spring leaf phenology than G_CC_ for trees with red spring leaves, we used R_CC_ metrics for the two trees where the spring amplitude in R_CC_ was greater than 40% of spring amplitude in G_CC_, and G_CC_ metrics for all other trees. We found that SOS was closest to 10% budburst (RMSD 4.7 days), although the date of 10% budburst was not significantly correlated to SOS across trees (r = −0.04, *p* > 0.05). This low (and not statistically significant) correlation is likely related to the fact that there was relatively little variability in the timing of budburst across trees: all trees under observation had 10% budburst within a period of about a week (DOY 121-129), while SOS date uncertainties were 11 days on average (inner 95% confidence interval, i.e., ±5.5 days). However, we found that our determinations of the first observable leaves in aerial image orthomosaics were correlated with SOS (r = 0.46, *p* < 0.05), indicating SOS dates derived from color-based vegetation indices were associated with biologically meaningful phenological transitions at the branch-to-canopy level.

**Figure 3:**
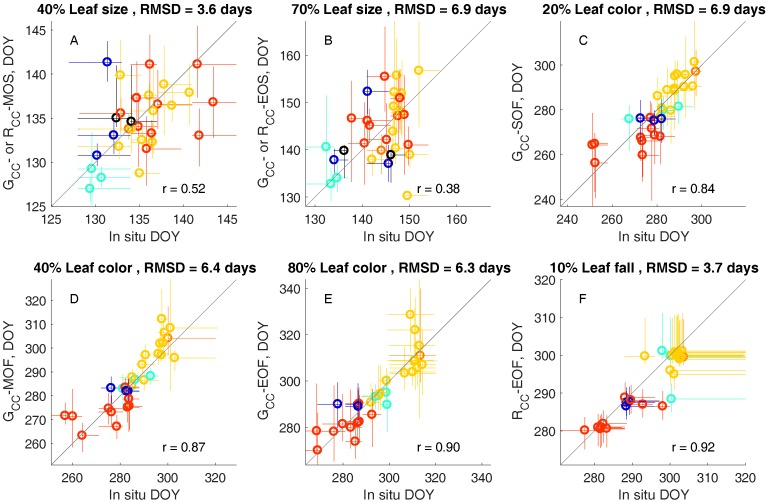
(**A**–**F**) leaf life cycle events corresponding to drone image metrics for 30 trees. Symbols denote transition dates, with confidence intervals shown as colored lines. Yellow = red oak (except trees with red spring leaves, shown in black in (**A**,**B**)), red = red maple, light blue = American beech, dark blue = yellow birch, and orange = black oak. Deciles of in situ observations (i.e., 40% leaf size) were selected as having the minimum RMSD in comparison to drone image metrics. Jitter was added to data points that lay on the same date and the one-one line is shown in black. Correlation coefficients are shown on each panel (all *p* < 0.05).

We found that MOS was closest to 40% leaf size (RMSD 3.6 days), and EOS was closest to 70% leaf size (RMSD 6.9 days; spring time G_CC_, budburst, and leaf size observations with associated transition dates shown in Figure S5 for an example tree). Later spring transitions were more variable across trees, with a range of 14 days for 40% leaf size and 18 days for 70% leaf size (Figure 3A,B). Across species, the two most common species, red oak and red maple, had similar leaf expansion phenology. However, American beeches were among the first trees to make the MOS and EOS transitions, and attain a 40% and 70% leaf size. The mean inner 95% confidence intervals across trees were six and 12 days for MOS and EOS metrics, respectively. MOS is typically the most certain metric in sigmoid curve fitting analyses [18] due to the clear timing of the steepest part of the increase in spring time G_CC_ (Figure 2M). Consequently, we found that the MOS from image analysis could represent tree-to-tree variation in the timing of leaf expansion observed in situ with greater statistical significance than EOS (MOS r = 0.52, *p* = 0.003; EOS r = 0.38, *p* = 0.04, Figure 3A,B).

To examine how representative color indices are of the progression of leaf life cycles at the tree level, we calculated HAC regressions between percentages of life cycle completion and index values across observation dates. Leaf size observations were more highly correlated with G_CC_ values in spring (average r = 0.95, 25 of 28 trees *p* < 0.01, Table S1) than budburst observations (average r = 0.86, 21 of 28 trees *p* < 0.01). We also note that, pooling across all trees with green spring leaves, G_CC_ values were highly correlated with leaf size observations (r = 0.88, *p* < 0.001), and to a similar but lesser extent, with budburst observations (r = 0.80, *p* < 0.001). However, we note that there were significant differences in regression slopes and intercepts between individual trees, even within species, according to *t*-tests (*p* < 0.05). This indicates that although there is a clear, statistically significant relationship between color indices and leaf life cycle progressions when all trees are pooled, there is also significant tree-to-tree variability in these relations when regression parameters are allowed to vary by tree.

For the two trees with red spring leaves, R_CC_ increased, and then declined in spring as leaves continued to expand and change in color from red to green (Figure 2F,G). Therefore, we considered R_CC_ values up to and including the spring maximum when examining correlations. Similar to G_CC_ for trees with green spring leaves, we found a higher correlation of R_CC_ with leaf size (Table S2, average r = 0.95, both trees *p* < 0.001) than budburst (average r = 0.76, neither tree *p* < 0.001). These results indicate that G_CC_ and R_CC_ values are more representative of the progression of leaf expansion than budburst, as these color indices are still increasing after the completion of budburst (e.g., Figures S2, S3 and S5).

The timing of leaf coloration events in autumn varies more among trees than either budburst or leaf expansion (Figure 3C–E) with a range of roughly 50 days in leaf color deciles across the 30 trees under observation. Species differences become more apparent in fall than in spring, with red oaks senescing after red maples, and American beeches and yellow birches in between these two. The SOF, MOF, and EOF transitions calculated from G_CC_ are fairly evenly spaced throughout the period of leaf color change, corresponding to 20% (RMSD 6.9 days), 40% (RMSD 6.4 days), and 80% (RMSD 6.3 days) leaf color, respectively. R_CC_-EOF, representing the date of the maximum R_CC_ value in autumn, was closest to 10% leaf fall (RMSD 3.7 days), the earliest abscission metric from in situ observations (Figure 3F).

G_CC_ values in autumn were negatively correlated with leaf color percentages (average r = −0.92, 26 of 30 trees *p* < 0.01, Table S1). While G_CC_ for deciduous trees generally declines as leaves senesce in autumn, R_CC_ increases to a maximum before declining to the dormant season level. We distinguished between these increasing and decreasing phases when examining correlations between R_CC_ and autumn phenological processes. R_CC_ values up to and including the fall maximum correlated with leaf color percentages (average r = 0.94, all trees *p* < 0.01, Table S3), while fall R_CC_ values including the maximum and later values were correlated with leaf fall percentages (tree average r = −0.94, 9 of 16 trees with sufficient observations *p* < 0.01). Similar to the springtime analysis, we found significant correlation when pooling all trees: leaf color was correlated to G_CC_, which decreased in autumn (r = −0.89, *p* < 0.001), as well as R_CC_ as it increased to the fall maximum (r = 0.84, *p* < 0.001). Leaf fall was more highly correlated to R_CC_ as it decreased after the fall maximum (r = −0.89, *p* < 0.001) than G_CC_ (r = −0.56, *p* < 0.001), since G_CC_ decreased due to color change, before leaves began falling. As in spring, we noted significant differences in regression slopes and intercepts between trees of the same species in these comparisons.

### 3.3. Linking Leaf Area to Color Indices

**Figure 4:**
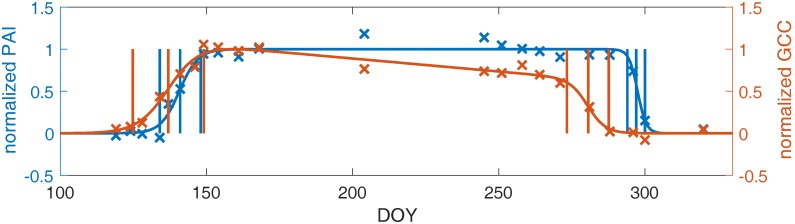
Smoothed (curve) and raw (× symbols) PAI and G_CC_ curves for an example microsite, and the dates derived from each data source (vertical lines show the SOS, MOS, EOS, SOF, MOF, and EOF dates). Values are normalized to have minimum 0 and maximum 1 according to the curve fits, to facilitate the comparison of PAI and G_CC_ trajectories.

We derived transition dates (SOS, MOS, etc.) from PAI time series, as well as from drone imagery using ROIs corresponding to the top-of-canopy area analyzed for PAI (example microsite shown in Figure 4). This allowed us to examine the relation between canopy structural development and canopy color development in drone images. The timing of the spring increase in PAI and G_CC_ was similar across the eight microsites, particularly for later spring transitions. At the first spring transition, SOS, the average difference between PAI and G_CC_ derived dates was −4.1 (negative sign indicates G_CC_-MOS was earlier) ±3.2 days standard deviation, and was significantly different from zero according to a paired *t*-test (*p* < 0.05). However, the MOS and EOS transitions were closer in time, with −0.6 ± 2.5 days and 2.6 ± 4.6 days differences, respectively. Both of these differences were not significantly different from zero. In autumn, declines in G_CC_ substantially preceded declines in PAI, as leaves changed color, becoming less green, before they were shed, decreasing the canopy PAI. As a result, PAI and G_CC_ transitions were all significantly different in autumn, with average differences of 12–15 days.

**Figure 5:**
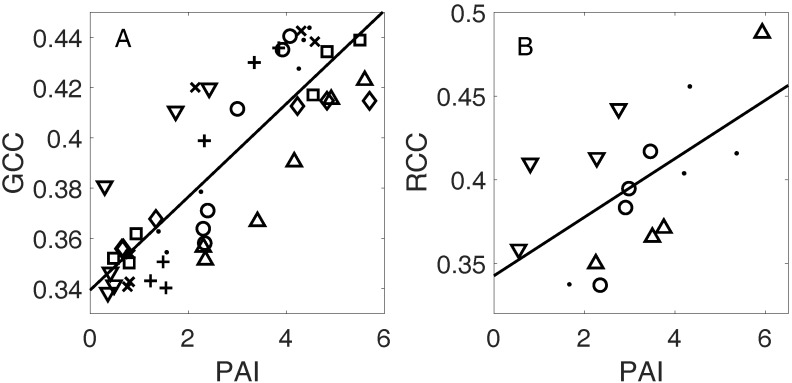
Scatter plots and lines of best fit between PAI values from upward photography and (**A**) G_CC_ values from drone imagery in spring (N = 48, eight microsites); (**B**) R_CC_ values after the maximum R_CC_ in fall, for microsites with one or more observations between the fall maximum of R_CC_ and the last observation (N = 16, four microsites). Different microsites are indicated by distinct symbols. The pooled-data regression equations are G_CC_ = 0.34 + 0.019*PAI (r = 0.84, *p* < 0.001) and R_CC_ = 0.34 + 0.017*PAI (r = 0.60, *p* < 0.05). Microsite-level correlations reported in Table S4.

To further investigate the connection between color indices and the canopy leaf area, we calculated the correlation of PAI to G_CC_ and R_CC_ values, pooled across microsites (Figure 5), as well as within microsites (Table S4). Spring G_CC_ was highly correlated to PAI (*p* < 0.001) when pooling all the data, as well as at individual microsites (*p* < 0.001 for six of eight microsites). However, there were significant differences in regression slopes and intercepts between microsites (*t*-test with *p* < 0.05). This is partially due to the fact that maximum PAI values vary more than maximum G_CC_ values, spatially throughout the forest. The maximum summertime PAI values we observed at the microsites varied from 3.4 to 6.4, while maximum G_CC_ varied from 0.44 to 0.48 (Table S4).

In fall, we examined the correlation between PAI and R_CC_ values from the fall R_CC_ maximum onward, following our observation that the peak of autumn R_CC_ indicates the beginning of leaf fall, which also marks the beginning of the decrease in the canopy leaf area. As in spring, we found evidence for linear correlation in pooled data across microsites, although there was a lower statistical significance (*p* < 0.05), likely due in part to a smaller sample size. Similarly, site-level correlations of R_CC_ and PAI were lower than in spring (average r = 0.90 versus 0.96 for springtime G_CC_ and PAI), although half of the microsites (two of four) with sufficient observations for analysis had a statistically significant linear correlation at *p* < 0.05 (based on ordinary least squares regressions, as the HAC adjustment for serial autocorrelation could not be used due to the small sample size).

### 3.4. Microclimate Effect on Phenology

To examine landscape variability in phenology, we calculated MOS and MOF dates of 10 m grid cells (Figure S6). We used G_CC_-MOS, except for six grid cells with a spring R_CC_ amplitude greater than 40% of the spring G_CC_ amplitude, based on the results of Section 3.1, and where we observed red spring leaves. We also filtered these grid cells to require the spring amplitude in R_CC_ to be greater than 0.02, to ensure that the color metric reflected a substantial change in redness that represented leaf growth as opposed to background noise. For these six grid cells, we used R_CC_-MOS. Variability across grid cells (Figure S6) was similar to that observed for these dates in in situ observations of trees (14 days range in MOS, 42 days in MOF, Figure 3), with a localized area of later spring and earlier fall phenology along a forest edge bordering a wetland, in the northwest of the study area (Figure S6).

We found that 29% of variance in MOS dates and 46% in MOF dates of 10 m grid cells was explained by spatial differences in the woody plant species assemblage (regression results in Table S5). Regression coefficients indicated a similar phenological order of species to that observed in situ: American beech was among the earliest species in spring, and red maple senescence preceded that of red oak by 23 days. In a previous analysis, we found evidence of spatial autocorrelation on length scales up to at least 30 m, in residuals from a similar regression. This was presumably due in part to fine-scale temperature variation [23]. Consequently, we examined residuals from the regression we performed here, in the 30 m by 30 m neighborhoods of the microsites where temperature measurements were made. Due to the low spatial variation in spring temperatures and a limited number of microsites, we did not detect significant relationships between microsite temperature and MOS residuals. In autumn, we found that the September mean of daily minimum temperature was correlated to the residuals of MOF dates (Bonferroni-adjusted *p* = 0.08, r = 0.77). We found an effect size of a five day delay (1.7 day SE) in MOF date per °C warmer (Figure S7).

## 4. Discussion

### 4.1. Leaf Life Cycles According to Image Metrics

#### 4.1.1. Exploration of Budburst, Leaf Growth, Color Change, and Fall

By making in situ observations of leaf life cycle events for individual trees, we were able to determine which phenology events corresponded to transitions identified from the color index (G_CC_ or R_CC_) analysis of images. Similar to Berra et al. [22], who also compared ROIs of individual trees in drone imagery to ground observations, and Ahrends et al. [9], who used individual tree ROIs from a tower-mounted camera, we found that observed budburst dates corresponded closely to the beginning of the increase in color indices in spring time, i.e., the SOS transition. But, while previous studies only focused on budburst, we also examined the relation of image metrics to in situ observations of leaf expansion, finding statistically significant correlations with this process as well. MOS and EOS were closest in time to 40% and 70% leaf expansion, respectively. Similarly, we found a higher correlation between springtime color indices and the leaf expansion process, than the progression of budburst. This indicates that while the initial increase in greenness (or redness) is a good indicator of the start of budburst, subsequent increases in index values are more representative of the leaf expansion process, as opposed to the completion of budburst.

We found that both G_CC_ and R_CC_ metrics were significantly correlated with leaf color change in autumn. These results varied somewhat by species as suggested by others [36], although we found that both G_CC_ and R_CC_ work reasonably well for all the species we observed. Red maples, which may display a variety of autumn colors from pale yellow to intense scarlet, had leaf color progressions that were more highly correlated to an increase in fall R_CC_ (species average r = 0.93) than a decrease in G_CC_ (−0.84). However, leaf color change in red oaks, which turn a yellow-orange color (Figure 2K), was similarly correlated to both G_CC_ and R_CC_ (species averages r = −0.96, 0.95, respectively). In terms of phenophase transition dates, we did not observe species-specific biases between in situ observations and dates calculated from image analysis (Figure 3).

Our results suggest that color indices may be used to describe the trajectories of both leaf color change and leaf fall in autumn, as well as key transition dates associated with these processes. Phenophase transitions calculated from G_CC_ (SOF, MOF, EOF) were highly correlated to a range of points in the progress of leaf coloration (20%, 40%, and 80% leaf coloration, respectively) across individual trees. The EOF metric determined from maximum fall R_CC_ was closest to the beginning of leaf fall, indicating that the greatest “redness” of trees occurs just as leaves are starting to abscise, although the redness of individual leaves may continue to increase after this [37]. Finally, a decline in R_CC_ after the autumn maximum was significantly correlated with the trajectory of leaf fall.

#### 4.1.2. Plant Area Index

Measures of the plant area index from an analysis of upward photos reinforce the results of the comparison of in situ observations with drone image metrics. We found significant correlations of PAI values with drone-derived G_CC_ in spring. This agrees with our result that in springtime, G_CC_ from drone images is associated with leaf expansion, which increases PAI. In general, the PAI time series and G_CC_ time series have similar trajectories in springtime (Figure 4 and Figure 5). Previous research using G_CC_ from tower-mounted cameras showed that the oblique angle of the camera field of view caused an early bias in late spring phenophase transitions relative to leaf area index (LAI, closely related to PAI) measured by the LAI-2000 instrument [19]. However, the nadir perspective of drone orthophotos appears to alleviate this effect; we found evidence for a strong linear relationship between springtime G_CC_ and PAI (Figure 5A).

We also found that the decrease in R_CC_ after the autumn maximum was significantly linearly correlated to the decrease in autumn PAI. This agrees with the earlier result that leaf fall, which decreases PAI, is correlated with R_CC_ at the individual tree level. Overall, our comparisons with in situ observations demonstrate the utility of drone image analysis for a thorough description of spring and autumn leaf phenology, including budburst, leaf expansion, senescence, and abscission.

### 4.2. Leaf Color and Color Indices: Red Spring Leaves

We observed a subpopulation of red oak trees that expressed reddish colors in young leaves. When isolating these trees for analysis, we found that a greenness index typically used in spring phenology studies of digital camera images (G_CC_) was not an effective characterization of leaf expansion phenology; however, a redness index was (R_CC_, Figure 2). Subsequently, we chose to use a combination of R_CC_ to describe the few trees or grid cells with spring amplitude in R_CC_ greater than 40% of spring amplitude in G_CC_, and G_CC_ for all other ROIs. While this criterion was useful in our identification of trees and grid cells with red spring leaves, we do not recommend it as a general rule since digital image color index values may vary by camera according to different image sensor characteristics [25], and different trees may have varying degrees of springtime redness. Researchers who observe red spring leaves should identify an appropriate criterion for their analysis.

We examined other options for color indices, including GRVI [38], ExG [17], and Hue [39]; however, these all had similar trajectories to G_CC_, which failed to describe leaf expansion phenology for trees with red spring leaves (Figure S2). We also calculated an index as the sum of G_CC_ and R_CC_ (equivalent to 1 minus blue chromatic coordinate, defined similarly to R_CC_ and G_CC_ [11]), in an attempt to combine the relevant information for trees with green and red spring leaves into a unified approach. However, because of the relatively large amount of noise in this index, it was less correlated to in situ observations of leaf expansion, and was less reliable in the 10 m grid cell analysis (caption to Figure S2).

While we found utility in using R_CC_ to quantify the phenology of trees with red spring leaves, to our knowledge, red leaves have not presented an issue for the many recent phenology studies using greenness indices to study spring phenology at Harvard Forest and elsewhere, e.g., [9,10,19,25]. This is presumably because of the relatively small number of trees with red spring leaves in relation to those with green spring leaves (i.e., only two of the 30 trees we observed in situ), and the fact that most phenology studies using digital images typically use regions of interest that integrate several tree crowns over the canopy. Indeed, we did not experience any redness-related issues with springtime G_CC_ for the larger ROIs we used to integrate over several trees for comparison to PAI measurements (533 m^2^ compared to individual tree ROIs, which were 31 m^2^ on average).

However, individual tree ROIs have recently been increasingly examined due to the advent of new methods in photographic studies of phenology. In crowd-sourcing [36], the time-intensive task of drawing regions of interest around many individual trees is alleviated by distributing it among many citizen scientists. In photogrammetry studies using drone imagery [22], larger study areas and an undistorted nadir view of the canopy facilitate the delineation of more individual tree crowns in imagery. As greater numbers of individual trees are analyzed, our results indicate that alternatives to greenness indices, such as the redness index we used here, should prove useful in identifying and analyzing trees with red spring leaves. We also note that besides color indices, texture analysis may yield useful approaches to phenological studies of forest canopies using digital images, as suggested in a recent exploratory study [40]. Texture analysis presents the possibility of color-independent image metrics, which may be applicable across trees with different leaf colors.

Red leaf color is more typically associated with autumn than spring in temperate forests, and is known to be associated with increased concentrations of anthocyanin pigments. However, a study on tropical trees indicated that young, immature leaves were more likely than senescent leaves to express a red color and increased anthocyanin concentrations, across different species [41]. Recent research indicated that, across diverse climates including temperate forests, young leaves of many tree and shrub species have a reddish appearance [42], including red oaks [43]. The physiological function of anthocyanins has not been settled; they have been hypothesized to serve a photoprotective role, as senescent leaves resorb nutrients [44], and may also benefit young, developing leaves in this way [41]. However, we observed differences in spring leaf color in conspecific individuals in close physical proximity and with a similar canopy position (Figure 2), suggesting that light microclimate may not be a determining factor. Our results indicate that drone photography would be a useful method for the further study of intraspecific differences in springtime leaf color.

### 4.3. Spatial Variance in Phenology and Relation to Microclimate

The effect of spatial temperature variation on phenology is most commonly explored over altitudinal gradients in mountainous terrain, in “space for time” studies that seek to use these gradients as a proxy for global change [45], or across large geographic distances [18,46]. However, temperature variation may also act on phenology over smaller spatial scales, due to microclimates induced by topography or canopy cover within forests [47,48]. We explored the effect of microclimate by first accounting for species composition effects, as variability in phenology among tree species is well known [26,49], and examining the relation of the residual phenological variance with air temperature.

Although we did not detect an effect of microsite temperature on spring phenology, we found evidence that warmer daily minimum temperatures in September delay senescence by five days per °C. These results agree with the chilling degree days hypothesis, which states that senescence is triggered when accumulated cold temperatures reach a critical threshold in autumn: colder temperatures advance senescence locally [31,46]. We note that microclimates with lower daily minimum temperatures were located along a forest edge, near a wetland to the northwest of the study area (Figure 1). This result highlights the importance of considering forest structure effects on microclimate and phenology [48], in addition to factors such as canopy position and tree developmental stage [26] when assessing the causes of phenological differences between trees.

## 5. Conclusions

We presented the first synthesis of in situ observations of tree phenology over a complete growing season with an analysis of drone images of the same trees. Using digital image processing techniques, we found that greenness metrics integrating several trees were correlated with canopy structural development (PAI) in springtime, while redness metrics were correlated with PAI decrease in autumn. At the individual tree level, we determined that the onset of rising greenness in spring corresponded with the start of budburst, while continued increases in greenness were driven by leaf expansion. However, we documented intraspecific variation in spring leaf color, noting that some oak trees displayed reddish (orange, pink) leaves during leaf-out while other trees had green leaves. We found that this affected the color index (greenness versus redness) that could be used to describe the leaf expansion phenology of individual trees. In autumn, we found that the decrease of canopy greenness, as well as the increase of redness, were correlated with the percentage of leaves that had changed color on trees. The time of greatest canopy redness corresponded to the beginning of abscission, and the trajectory of leaf fall correlated with decreasing redness. These results leverage the novel method of drone photogrammetry to advance our understanding of how digital image metrics relate to foliar phenology and canopy structural development throughout the leaf life cycle, from budburst to abscission.

## Supplementary Materials

The following are available online at http://www.mdpi.com/1424-8220/17/12/2852/s1.
