# Supplementary material for: Observing Spring and Fall Phenology in a Deciduous Forest with Aerial Drone Imagery
Source: Sensors (Basel). 2017 Dec 8;17(12):2852. doi: 10.3390/s17122852 (PMC5751649; doi:10.3390/s17122852)
Supplement: Supplementary file 1 [file sensors-17-02852-s001.pdf]

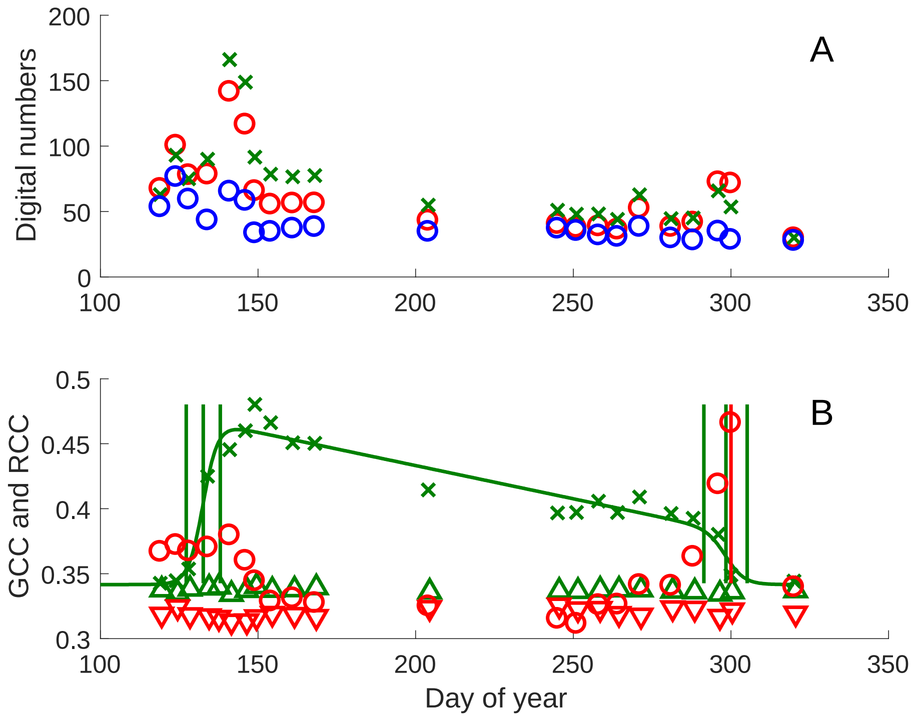

Figure S1. **(a)** Raw data for an example 10 m grid cell: average digital numbers of red, green, and blue bands calculated from an orthophoto image file, shown as circle symbols (R, B) and 'x' symbols (G) in those colors, and **(b)** the resulting Gcc and Rcc values (green 'x' and red circle symbols, respectively); Gcc curve fit (green curve); estimated dates for start, middle, and end of spring and fall from Gcc (vertical green lines) and end of fall from Rcc (vertical red line); and Gcc and Rcc values of a gray reference square (green upward-pointing triangles and red downward-pointing triangles, respectively). We added a Gcc data point on DOY 50 (not shown), equal to the average of the first and last observed data points for the year, to provide additional spring baseline and reliable date estimates (e.g. [46]). The amplitudes of the seasonal signals of Gcc and Rcc in vegetation are approximately 10 times greater than the noise from the reference square (ranges of reference square values are 0.006 for Gcc, 0.012 for Rcc). Reference square noise can be attributed to, for example, changes in scene illumination between dates, due to varying sky conditions, and different times of day of flights on different dates.

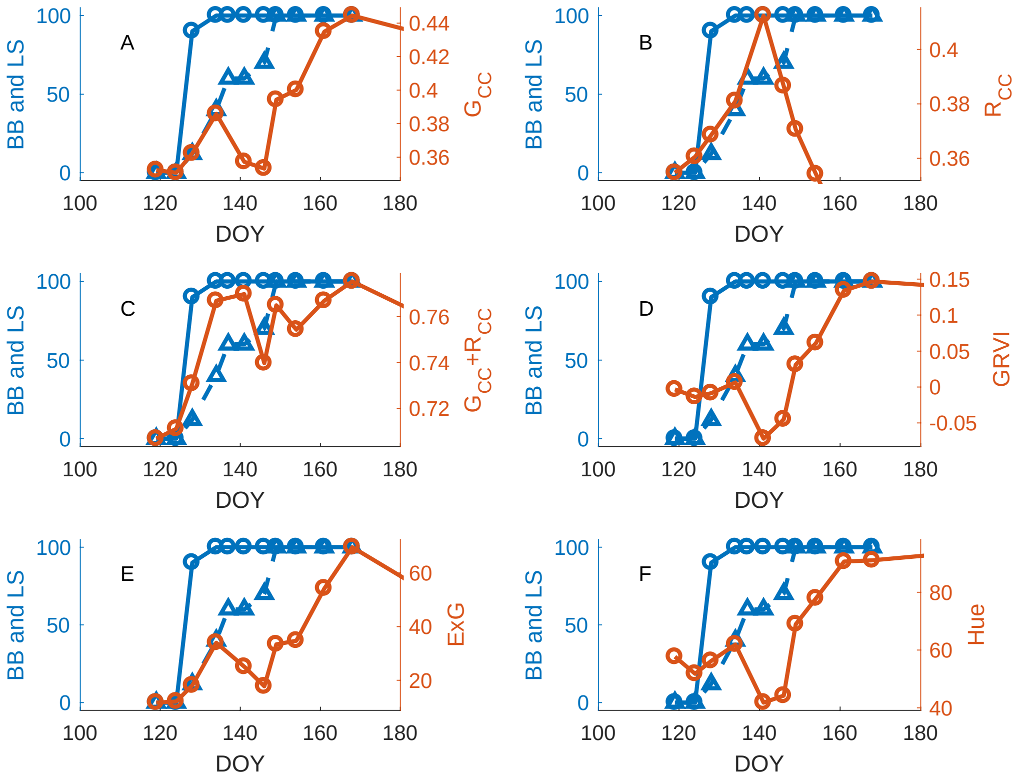

Figure S2. Spring time color indices (orange lines with circles), budburst trajectories (blue solid lines with circles), and leaf size trajectories (blue dashed lines with triangles), for the tree with red spring leaves shown in Figure 2 (tree ID 331029). **(a)** G_{CC}, from Equation 1. **(b)** R_{CC}, from Equation 2. **(c)** G_{CC} + R_{CC}. **(d)** GRVI = (G – R)/(G + R), [38]. **(e)** ExG = 2G – (R + B), [17]. **(f)** Following Nagai et al. [39],

$$Hue = \begin{cases} 60 \times (b - g)\ if\ R = \max(DN) \\ 60 \times (2 + r - b)\ if\ G = \max(DN) \\ 60 \times (4 + g - r)\ if\ B = \max(DN) \end{cases} \qquad \text{(S1)}$$

where max(DN) = max{R, G, B}, min(DN) = min{R, G, B}, r = (max(DN) – R)/(max(DN)-min(DN)), g = (max(DN) – G)/(max(DN)-min(DN)), and b = (max(DN) – B)/(max(DN)-min(DN)). If Hue < 0, we added 360 to it.

We note that GRVI, ExG, and Hue follow the same pattern as G_{CC}, exhibiting a slight initial increase after budburst and then a dip during leaf expansion. R_{CC} corresponds very closely to leaf expansion. G_{CC} + R_{CC} has a marked increase, closer in time at 50% amplitude to leaf size for this tree, than all other indices besides R_{CC}.

G_{CC} + R_{CC} also presents the possibility of using one index for all trees as it represents changes in both greenness and redness. However, because of this mixing of signals, G_{CC} + R_{CC} time series are noisier, as shown here. We found that the MOS transition estimated from G_{CC} + R_{CC} was closest in time to 20% leaf size across trees; however, the correlation was $r = 0.44$, $p > 0.01$, compared with the combination of G_{CC} for most trees and R_{CC} for those with red spring leaves (which was closest to 40% leaf size, Figure 3A), which was $r = 0.52$, $p < 0.01$ ($n = 30$ trees). Additionally, the curve fitting algorithm failed to generate dates for more 10 m grid cells using the G_{CC} + R_{CC} index (12%) than the combination of either G_{CC} or R_{CC} (5%), presumably due to a lower signal to noise ratio. Therefore, we used either G_{CC} or R_{CC} as described in sections 3.1 and 3.4.

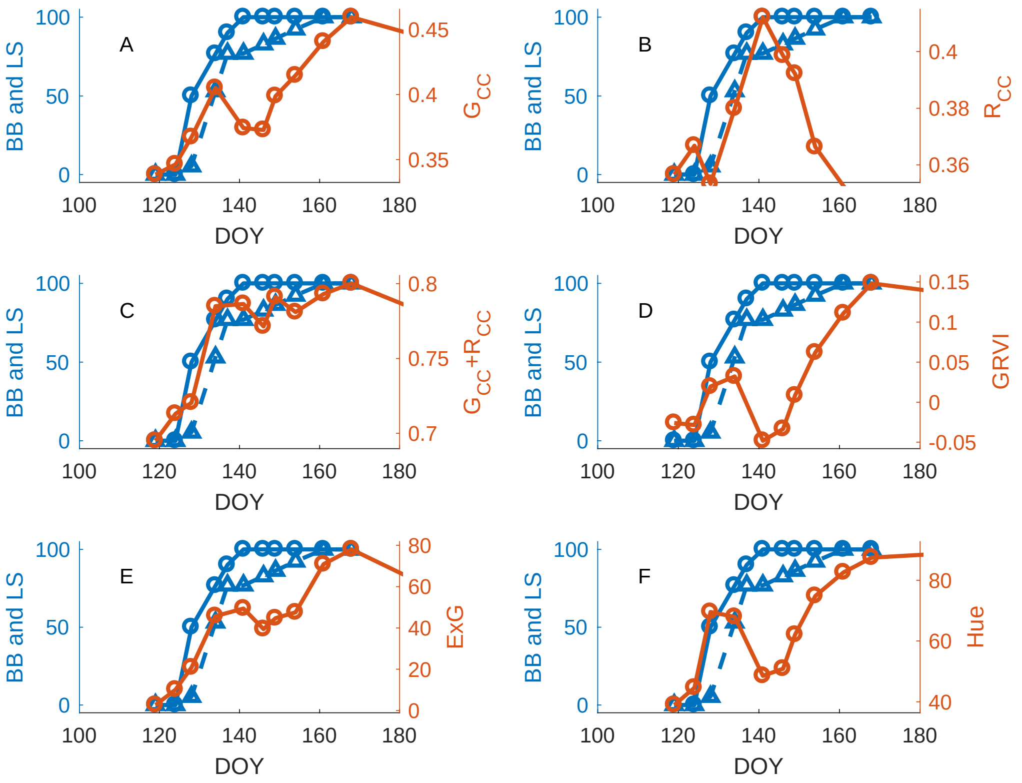

Figure S3. Similar to Figure S2, but for tree ID 311817, the other red oak tree under observation that had red spring leaves.

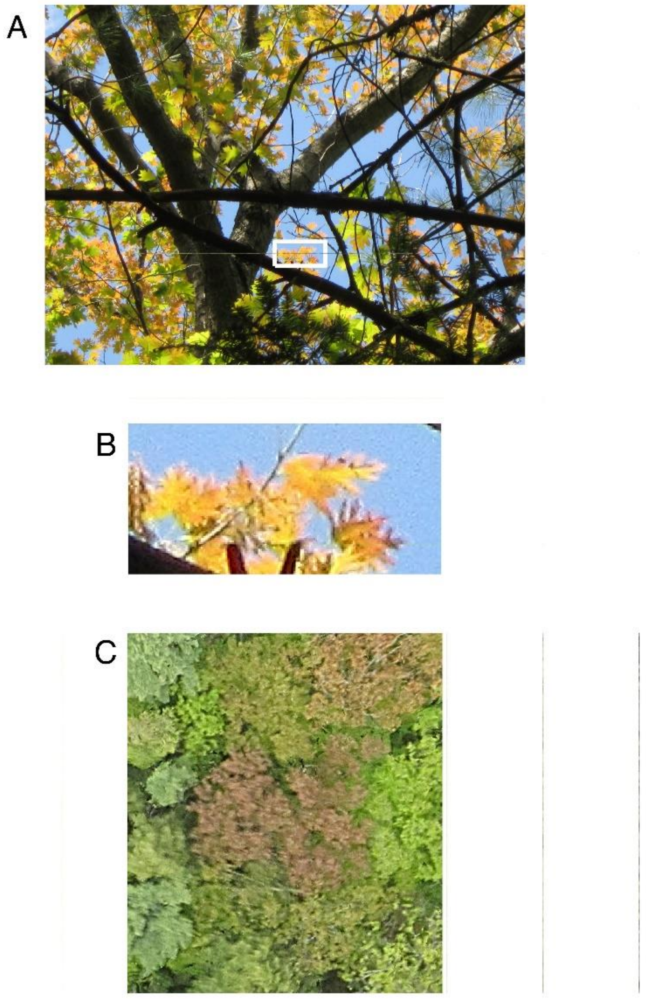

Figure S4. (a) Photo from the ground of a red oak tree with red spring leaves, on May 17, 2015, with **(b)** closeup of red leaves from inset area, and **(c)** aerial photo of the same tree four days later on May 21, 2015. Leaves higher in the canopy are seen to be red, while those closer to the ground are green. Sharpening filter applied to **(b)** and **(c)** to enhance clarity.

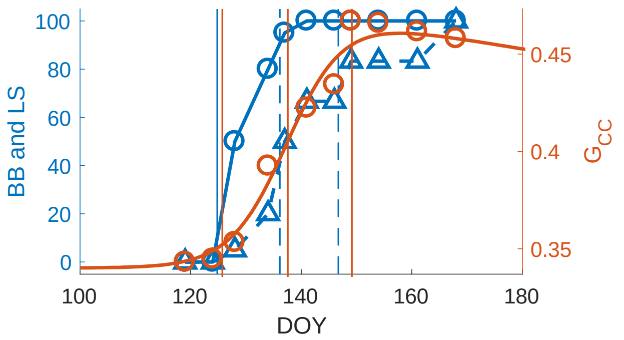

Figure S5. Springtime color index and in situ observations for a tree with green spring leaves. Gcc (orange circles) with curve fit (orange line) and SOS, MOS, and EOS transition dates (vertical orange

lines); budburst trajectory (blue solid line with circles) with 10% budburst date (vertical blue solid line); and leaf size trajectory (blue dashed line with triangles) with 40% and 70% leaf size dates (vertical blue dashed lines).

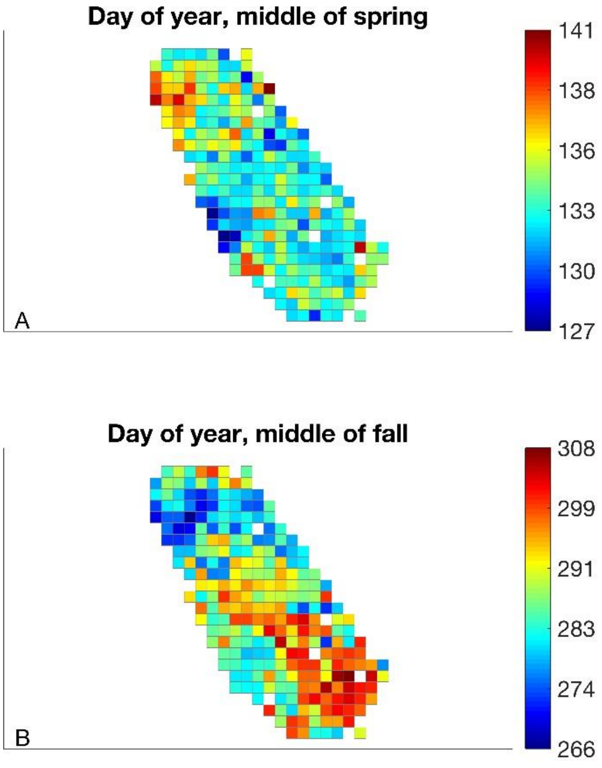

Figure S6. (**a**) MOS and (**b**) MOF dates of 10 m grid cells in the study area, by day of year. Grid cells shown in white failed to generate phenology dates, primarily owing to the small range in color indices due to evergreen vegetation.

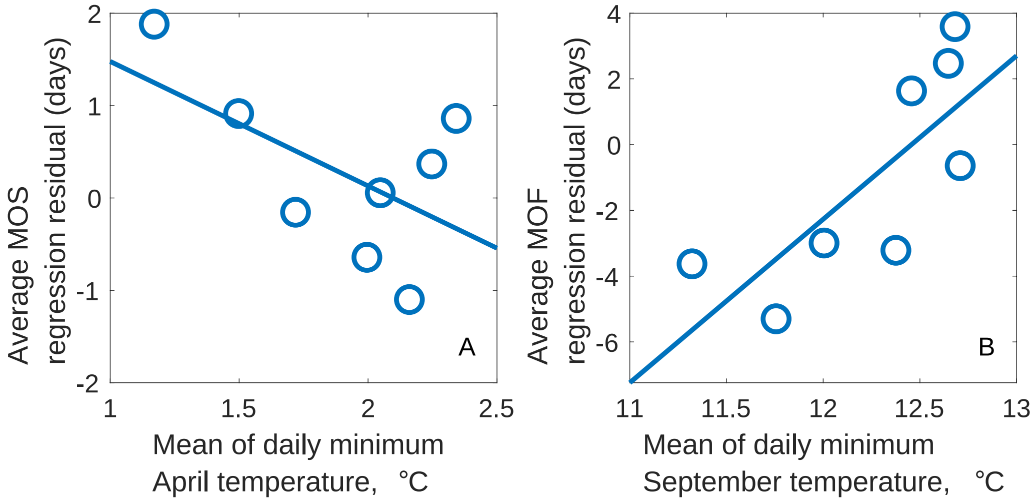

Figure S7. Scatter plots and lines of best fit for average daily minimum temperatures and average regression residuals at microsites: **(a)** April temperatures with MOS residuals and **(b)** September temperatures with MOF residuals. Residuals are calculated from a regression of phenology dates on

species composition, averaged over the three by three windows of grid cells (30 m by 30 m window area) around each temperature measurement location. Positive residual indicates a later observed transition than predicted. Warmer temperatures generally correspond to earlier spring transitions than predicted by species alone, although due to the limited sample size and temperature variability, the spring regression is not significant (r = -0.57, Bonferroni-adjusted p = 0.4). Warmer temperatures are correlated with later fall predictions (r = 0.77, Bonferroni-adjusted p = 0.08). Bonferroni adjustments were made to account for the three temperature comparisons (average daily minimum, maximum, and mean); the most highly correlated comparisons are shown for each phenology transition.

Table S1. Correlation coefficients and HAC-adjusted p-values for G_{CC} values and percentages of budburst (BB), leaf size (LS), leaf color (LC), and leaf fall (LF) on individual trees. Species codes use the first four letters of the genus name and first two letters of the species name. N = 10 for all correlations. Significance levels are indicated as *: p < 0.05, **: p < 0.01, ***: p < 0.001.

		BB		LS		LC		LF	
**Species**	**Tree ID**	**r**	**p**	**r**	**p**	**r**	**p**	**r**	**p**
acerru	281280	0.83	9.5E-03**	0.94	4.7E-05***	-0.97	1.1E-05***	-0.73	2.4E-02*
querru	281278	0.90	3.7E-03**	0.98	4.7E-04***	-0.97	7.8E-09***	-0.7	4.8E-04***
acerru	281357	0.92	2.8E-03**	0.91	4.7E-03**	-0.87	7.6E-04***	-0.6	1.2E-01
querru	291458	0.85	1.3E-02*	0.95	5.3E-03**	-0.94	1.9E-08***	-0.47	7.6E-04***
betual	291485	0.77	1.5E-02*	0.99	1.8E-05***	-0.92	3.8E-06***	-0.72	7.7E-03**
acerru	291500	0.94	1.2E-03**	0.91	2.3E-04***	-0.71	3.1E-02*	-0.23	2.5E-01
acerru	282598	0.90	3.6E-03**	0.92	3.1E-03**	-0.81	4.0E-03**	-0.48	1.6E-01
querru	282517	0.91	3.7E-03**	0.98	1.2E-04***	-0.97	1.0E-09***	-0.31	6.2E-03**
acerru	282593	0.88	7.6E-03**	0.96	5.5E-04***	-0.77	1.3E-02*	-0.12	3.3E-01
fagugr	311754	0.87	3.2E-03**	0.94	3.5E-04***	-0.99	2.4E-07***	-0.51	1.4E-03**
fagugr	311822	0.90	1.2E-03**	0.95	2.1E-04***	-0.92	9.2E-04***	-0.46	1.1E-02*
fagugr	311821	0.86	1.2E-03**	0.97	3.3E-04***	-0.84	3.6E-03**	-0.28	1.7E-02*
querru	311817					-0.95	1.7E-07***	-0.49	1.9E-04***
querru	301810	0.94	1.4E-03**	0.9	8.3E-03**	-0.97	1.7E-10***	-0.73	3.6E-04***
querru	340761	0.72	1.8E-01	0.98	2.4E-04***	-0.98	7.8E-08***	-0.6	1.8E-03**
querve	350677	0.74	1.9E-02*	0.97	7.5E-04***	-0.97	2.9E-02*	-0.89	5.9E-07***
querru	350683	0.77	1.4E-02*	0.95	5.8E-02	-0.96	2.8E-11***	-0.88	3.7E-07***
querru	350736	0.84	7.5E-03**	0.97	2.5E-03**	-0.96	9.7E-09***	-0.75	2.1E-04***
querru	350633	0.81	8.3E-03**	0.96	2.0E-03**	-0.95	1.6E-07***	-0.87	1.7E-05***
acerru	341091	0.96	2.4E-04***	0.98	1.4E-04***	-0.79	9.2E-03**	-0.56	1.5E-01
betual	341090	0.85	7.5E-03**	0.93	3.0E-02*	-0.96	9.0E-07***	-0.67	2.5E-03**
acerru	341086	0.93	3.3E-03**	0.98	6.3E-05***	-0.78	1.1E-02*	-0.56	1.5E-01
querru	341108	0.85	6.6E-03**	0.90	3.4E-03**	-0.96	6.4E-05***	-0.86	7.4E-06***
querru	341109	0.80	2.7E-01	0.96	2.3E-04***	-0.96	5.5E-10***	-0.95	6.0E-07***
querru	331069	0.85	6.4E-03**	0.89	3.9E-02*	-0.98	1.0E-09***	-0.87	3.4E-06***
acerru	331003	0.96	4.3E-04***	0.90	8.9E-03**	-0.97	2.0E-08***	-0.69	1.9E-06***
querru	331029					-0.96	1.6E-08***	-0.80	5.8E-05***
acerru	310896	0.96	1.2E-03**	0.97	1.3E-03**	-0.87	4.5E-03**	-0.29	2.7E-01

betual	310895	0.70	2.3E-02*	0.95	1.2E-03**	-0.99	2.4E-09***	-0.77	3.5E-03**
acerru	310889	0.98	1.3E-04***	0.89	1.8E-03**	-0.82	9.8E-06***	-0.43	4.6E-02*
Average		0.86	2.2E-02*	0.95	6.1E-03**	-0.92	3.6E-03**	-0.61	5.1E-02

Table S2. Correlation coefficients and HAC-adjusted p-values for R_{CC} values up to and including the spring maximum, and percentages of budburst (BB) and leaf size (LS) on individual trees. Species and significance codes as in Table S1. N = 5.

		**BB**		**LS**	
**Species**	**Tree ID**	**r**	**p**	**r**	**p**
querru	311817	0.77	1.1E-03**	0.93	6.7E-05***
querru	331029	0.75	5.9E-03**	0.97	1.5E-04***
Average		0.76	3.5E-03**	0.95	1.1E-04***

Table S3. Correlation coefficients and p-values for R_{CC} values before and including the fall maximum with percentages of leaf color (LC), and after and including the fall maximum with leaf fall (LF) on individual trees. Species and significance codes as in Table S1. The HAC procedure could not be applied due to the small sample size in the comparison of R_{CC} and leaf fall; these are ordinary least squares regressions and may have underestimated p-values due to serial autocorrelation.

		**LC**			**LF**		
**Species**	**Tree ID**	**r**	**p**	**N**	**r**	**p**	**N**
acerru	281280	0.97	7.7E-05***	6	-0.72	1.7E-01	5
querru	281278	0.95	1.8E-06***	9			2
acerru	281357	0.98	4.1E-05***	6	-0.97	5.3E-03**	5
querru	291458	0.87	4.1E-05***	8	-1.00	4.8E-02*	3
betual	291485	0.87	4.0E-06***	7	-0.93	7.3E-02	4
acerru	291500	0.82	2.8E-03**	6	-1.00	2.5E-04***	5
acerru	282598	0.90	1.2E-05***	7	-0.98	1.7E-02*	4
querru	282517	0.95	2.4E-06***	8	-0.99	9.6E-02	3
acerru	282593	0.95	1.1E-05***	6	-0.99	5.4E-04***	5
fagugr	311754	0.97	1.3E-04***	7	-1.00	2.7E-03**	4
fagugr	311822	0.87	2.2E-03**	9			2
fagugr	311821	0.91	5.6E-04***	9			2
querru	311817	0.98	7.8E-07***	9			2
querru	301810	0.98	4.1E-07***	9			2
querru	340761	0.99	3.1E-09***	9			2
querve	350677	0.93	2.4E-05***	9			2
querru	350683	0.98	8.6E-07***	9			2
querru	350736	0.98	7.1E-07***	9			2
querru	350633	0.96	1.2E-03**	9			2
acerru	341091	0.79	8.6E-03**	6	-0.78	1.2E-01	5
betual	341090	0.97	2.9E-06***	7	-1.00	1.1E-03**	4
acerru	341086	0.89	4.0E-05***	6	-0.95	1.5E-02*	5
querru	341108	0.95	9.3E-07***	9			2
querru	341109	0.93	5.7E-06***	9			2

querru	331069	0.98	7.0E-07***	9			2
acerru	331003	0.99	3.4E-07***	7	-0.87	1.3E-01	4
querru	331029	0.93	1.1E-06***	9			2
acerru	310896	1.00	1.5E-10***	7	-0.79	2.1E-01	4
betual	310895	0.97	6.8E-07***	7	-1.00	6.6E-04***	4
acerru	310889	0.97	2.1E-07***	7	-1.00	4.0E-03**	4
Average		0.94	5.3E-04***	7.8	-0.94	5.6E-02	3.2

Table S4. Correlation coefficients and p-values for Gcc and PAI in spring, and Rcc after and including the fall maximum and PAI in fall, at each of the eight microsites, as well as the maximum summer PAI and Gcc at each microsite. The HAC procedure could not be applied due to the small sample size in the comparison of Rcc and PAI; these are ordinary least squares regressions and may have underestimated p-values due to serial autocorrelation. N = 6 in spring and 4 in autumn. Significance codes as in Table S1.

	**Spring/Gcc**		**Fall/Rcc**			
**Microsite**	**r**	**p**	**r**	**p**	**Max PAI**	**Max Gcc**
1	0.97	2.1E-05***			5.1	0.46
2	0.98	1.3E-04***	0.99	1.4E-02*	4.5	0.46
3	0.87	4.3E-03**	0.83	1.7E-01	3.4	0.45
4	0.99	1.1E-04***	0.81	1.9E-01	5.5	0.48
5	0.94	5.9E-03**			5.7	0.47
6	0.99	4.9E-05***			5.9	0.47
7	0.98	3.4E-04***			6.3	0.44
8	0.98	1.0E-04***	0.95	4.5E-02*	6.4	0.45
Average	0.96	1.4E-03**	0.90	1.1E-01	5.3	0.46

Table S5. Regression coefficients and standard errors (SE) for the multiple linear regression of the middle of spring and fall dates of 10 m grid cells (n = 245). Predictors include a categorical variable for forest land cover type (as opposed to wetland, which consisted of the four grid cells along the western edge of the northern portion of the study area shown in Figure 1) and deciduous species composition by basal area fraction for the indicated species. Deciduous species are ordered by prevalence in terms of total basal area in all grid cells, and include red oak (*Quercus rubra*), red maple (*Acer rubrum*), yellow birch (*Betula alleghaniensis*), American beech (*Fagus grandifolia*), black cherry (*Prunus serotina*), black birch (*Betula lenta*), and winterberry (*Ilex verticillata*), and a lumped group of all other species. Spring coefficients for the top four deciduous species have the same rank order as we found in a previous study of 2013 phenology in this area [23], although were four to six days later for these 2015 results. Fall coefficients for these species were similar with an average difference of less than one day. Total sums of squares, model sums of squares, and RMSDs were 1356, 398, and two days for spring, and 19033, 8682, and seven days for fall, respectively.

**Variable**	**Spring coefficient**	**Spring SE**	**Fall coefficient**	**Fall SE**
Cover type forest	-4	1.1	6	3.5
Red oak	137	1.1	294	3.7
Red maple	140	1.1	271	3.6
Yellow birch	135	1.6	267	5.3

American beech	134	1.7	285	5.5
Black cherry	134	4.9	258	16.1
Black birch	134	3.0	293	9.9
Winterberry	147	3.6	277	12.0
Other	135	1.6	290	5.3
